# Bamboo shoots-derived nanovesicles (BSNs) induce apoptosis in non-small-cell lung cancer A549 cells through the p53 signaling pathway

**DOI:** 10.3389/fmolb.2026.1759968

**Published:** 2026-04-09

**Authors:** Ruihua Bai

**Affiliations:** Key Laboratory of High Efficient Processing of Bamboo of Zhejiang Province, China National Bamboo Research Center, Hangzhou, Zhejiang, China

**Keywords:** A549 cells, apoptosis, bamboo shoot, nanovesicle, non-small-cell lung cancer

## Abstract

**Introduction:**

Non-small cell lung cancer (NSCLC) remains a leading cause of cancer-related mortality worldwide. Nanovesicles have been demonstrated to be important mediators of intercellular communication in NSCLC. Plant-derived nanovesicles contain lipids, proteins, nucleic acids, and pharmacologically active substances, and have attracted increasing attention as potential anticancer agents due to their easy availability, low toxicity, and demonstrated biological activities. However, the role of edible plant-derived nanovesicles in cancer regulation remains poorly understood.

**Methods:**

Bamboo shoot-derived nanovesicles (BSN) were isolated by differential centrifugation and characterized. The human NSCLC cell line A549 was used as an *in vitro* model. Cellular uptake of BSN was evaluated, and their effects on cell proliferation, migration, and invasion were assessed. Selectivity was examined using non-cancer cells (3T3-L1 and RAW264.7) and another cancer cell line (HeLa). RNA sequencing (RNA-seq) was performed to explore the underlying molecular mechanisms, followed by protein-protein interaction (PPI) network analysis. Apoptosis and signaling pathways were further analyzed by flow cytometry and Western blot.

**Results:**

BSN were efficiently internalized by A549 cells and significantly inhibited A549 cell proliferation, migration, and invasion. This inhibitory effect was not observed in non-cancer cells (3T3-L1 and RAW264.7) or in another cancer cell line (HeLa). RNA-seq analysis indicated that BSN treatment mainly affected the cell cycle and p53 signaling pathways. Among the Top 20 core genes identified in the PPI network, 13 were associated with Akt-related pathways. Further experiments demonstrated that BSN promoted apoptosis in A549 cells through modulation of the Akt/p53 signaling pathway.

**Discussion:**

These findings indicate that BSN selectively target NSCLC cells and induce apoptosis via the Akt/p53 pathway, thereby exerting anti-tumor effects in A549 cells. This study highlights the potential of bamboo shoot-derived nanovesicles as a promising plant-based therapeutic strategy for NSCLC.

## Highlights

- Bamboo shoot-derived nanovesicles (BSNs) are identified as exosome-like nanovesicles that can be internalized by non-small cell lung cancer A549 cells.
- BSNs can inhibit the proliferation, migration, and invasion of A549 cells.
- RNAseq analysis revealed that BSNs enhance the expression of genes related to the cell cycle, ferroptosis, and p53 signaling pathways.
- BSNs can promote apoptosis in A549 cells by activating the p53 pathway, showing potential as an anticancer drug.

## Introduction

Among all cancers, lung cancer is one of the top-ranking killer cancers in China and worldwide (Chen et al., 2016; Ramalingam, Owonikoko and Khuri, 2011). Non-small cell lung cancer (NSCLC) occupies approximately 85% of the primary lung cancer, which in turn comprehends adenocarcinomas (ADC), squamous cell (SCC), and large cell carcinomas (LCC) based on histopathological heterogeneity (Chen et al., 2016). Nearly 80% of NSCLC patients present distal metastasis, causing poor prognosis and even death (Ramalingam et al., 2011). Currently, the clinical therapeutic strategy for lung cancer available to patients mainly includes surgical resection combined with chemotherapy and radiotherapy. However, postoperative complications adversely affect survival, such as post-operative adhesions and infection (Chen Z. et al., 2022; Dhall et al., 2019). Moreover, serious damage to the normal tissues was caused by the undesirable toxicity of conventional chemotherapy agents (Bai et al., 2023). Furthermore, long-term administration of chemotherapy agents may induce drug resistance (Taron et al., 2004). Therefore, to achieve better outcomes for NSCLC, the development of low-toxicity and proven efficacy anticancer agents in NSCLC is urgently needed.

The medicinal properties of plant extracts have never been in doubt as many extracts of the plant have been documented to possess antitumor, anti-infection, antioxidation, anti-inflammation, and immunoregulatory characteristics (Chang et al., 2017; Jongrungraungchok et al., 2023; Ramakanthet al., 2016; Xiong et al., 2018). In this aspect, the natural products of plant origin can serve as a large drug database for the research and development of anti-tumor agents with high efficiency, low toxicity, and cost-efficiency. Currently, many plant-derived antitumor drugs have been reported, such as curcumin which is believed to help prevent NSCLC (Kalwani et al., 2015), Quercetin which mediated HL-60 cell death (Kalwani et al., 2015), hydroalcoholic extract of H. purpurascens which induced the process of apoptosis in skin and breast cancer (Pilut et al., 2022), β-element which had a synergistic anti-hepatocellular carcinoma (HCC) effect with oxaliplatin (Li et al., 2016). Bamboo is an evergreen woody grass, that belongs to the Gramineae Bambusoideae and is widely distributed in China, Japan, and other East Asian countries (He et al., 2014). Bamboo shoots are immature edible stems on the rhizome nodes of bamboo and have been widely used as a medicinal and edible food or Chinese medicine (Chen C. et al., 2022). Previous studies have proved that bamboo shoot polysaccharides relieve Antibiotic-associated diarrhea in mice by gut microbiota regulation (Chen C. et al., 2022). Bamboo shoots with high dietary fiber content effectively reduced the development of obesity (Wu et al., 2020). Moreover, one of the bamboo shoot extracts, bamboo shoot dietary fiber-1(BSDF-1) is reported to exert an anti-inflammatory response in DSS-induced colitis through suppression of NF-κB pathway and NLRP3 inflammasomes (Li et al., 2022). Nevertheless, there is little information available about the characteristics and antitumor properties of bamboo shoot extract.

In this study, we showed that bamboo shoot extract contains nanoparticles with morphological and complete membrane systems, which allowed us to term them exosome-like nanovesicles. Here, bamboo shoots-derived nanovesicles (BSNs) were extracted and displayed an *in vitro* antineoplastic activity on A549 cells. Moreover, we confirmed that BSNs specifically inhibited the proliferation and migration, and suppressed cell invasion of lung cancer A549 cell lines. BSNs exerted their antitumor property by stimulating a p53-mediated apoptotic mechanism. Collectively, our findings inspired or provided a natural anticancer agent option, with low cost, high efficiency, and slow toxicity against NSCLC. Nanoparticles from natural substances suggested a feasible approach to eradicate cancer in the coming decades.

## Results

### Isolation and characterization of bamboo shoot–derived nanovesicles

**FIGURE 1:**
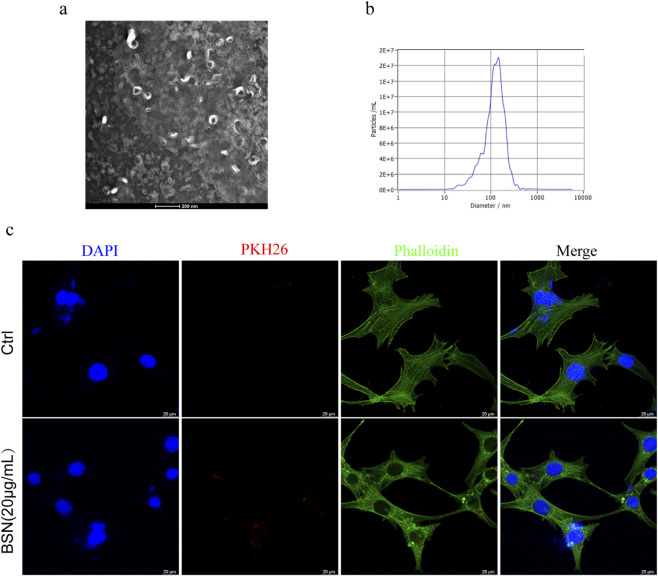
The characterization of BSNs. **(a)** The morphology of nanovesicles was observed by TEM. Scale bar: 200 nm. **(b)** Diameter distribution of BSNs detected by NTA (mm). **(c)** PKH26-labeled exosomes (red) visualized by confocal microscopy in A549 cell, blue indicated nuclei. Scale bar: 20 μm.

BSNs were isolated from Moso bamboo shoots. The integrity and morphology of nanovesicles were analyzed using transmission electron microscopy as shown in Figure 1a. We next carried out nanoparticle tracking analysis (ZetaView, *Particle Metrix*) and recorded median concentrations of 2.6 × 10^8^ particles/ml and an average size of 120.9 nm (Figure 1b). Studies have shown that plant-derived nanovesicles typically range in size from 50 to 200 nm (Sha et al., 2024; Yi et al., 2023). Based on the size and morphology, our data showed that BSNs are exosome-like vesicles.

### Cellular uptake of BSNs by A549 cells

We labeled isolated nanovesicles with PKH26, a lipophilic dye, to determine if BSNs are internalized by A549 cells. We observed that PKH26-labeled nanovesicles were internalized by A549 cells which stained with phalloidin-iFluor488 for cytoskeleton (Figure 1c), suggesting that the BSNs may be in the regulation of the biological function of A549 cells.

### Effects of BSNs on proliferation, migration, and invasion of A549 cells

**FIGURE 2:**
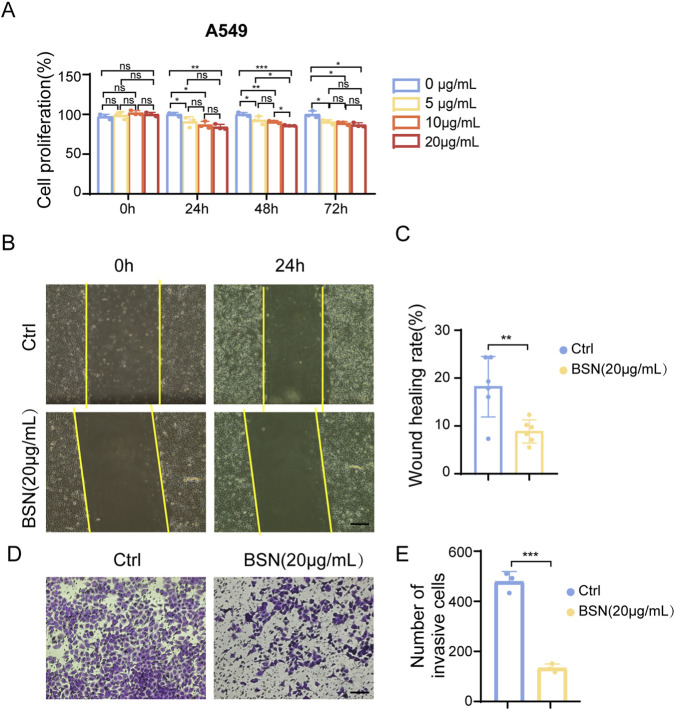
The therapeutic potent of BSNs in A549 lung cancer cells. **(A)** The proliferation rate of A549 cells under BSNs treatment was determined by CCK8 assays. **(B)** Scratch wound healing assay was performed to determine cell migration of A549 cells. Scale bar: 400 μm **(C)** Graphs showing quantification of wound healing rate. **(D)** Transwell migration assay was performed to determine the invasion ability of A549 cells. Scale bar: 400 μm **(E)** Graphs showing quantification of the number of invasion cells. All experiments were performed at least in triplicates, values are presented as mean ± SD. ^ns^P > 0.05, *P < 0.05, **P < 0.01, ***P < 0.001, ****P < 0.0001, compared with control.

To access the potential impact of BSNs on metastatic properties of lung cancer cells, their effects on the proliferation, migration, and invasion in lung adenocarcinoma A549 cells were examined in terms of CCK8 assay, scratch healing assay, and transwell assay. As expected, BSNs showed inhibition of A549 cell proliferation with a 5,10,20 μg/mL concentration gradient of BSNs at 24 h, 48 h, and 72 h after BSNs treatment (Figure 2A). It is noteworthy that the degree of inhibition was at dose dependence in the concentrations range (5–20 μg/mL) tested and persisted for at least 72 h (Figure 2A). To assess the specificity of BSNs against tumor cell lines, non-cancer cell line 3T3-L1 treated with same conditions. BSNs inhibited the viability of 3T3-L1 at various BSNs concentrations, whereas this consistency was not seen at the 48-h and 72-h time points (Supplementary Figure S1A). Inhibitory effects of BSNs were also observed in cell migration of A549, but not in another tumor cell line, Hela, and non-cancer cell line, 3T3-L1 and RAW264.7 (Figures 2B,C; Supplementary Figures S1B–E). In addition, BSNs also exhibited an ability to inhibit cell invasion in A549 cells as observed in the transwell assay at a concentration of 20 μg/mL (Figures 2D,E).

The results reported above showed that BSNs were active against A549 proliferation and metastasis with a specificity of their effect on human lung cells.

### Transcriptomic alterations induced by BSNs

**FIGURE 3:**
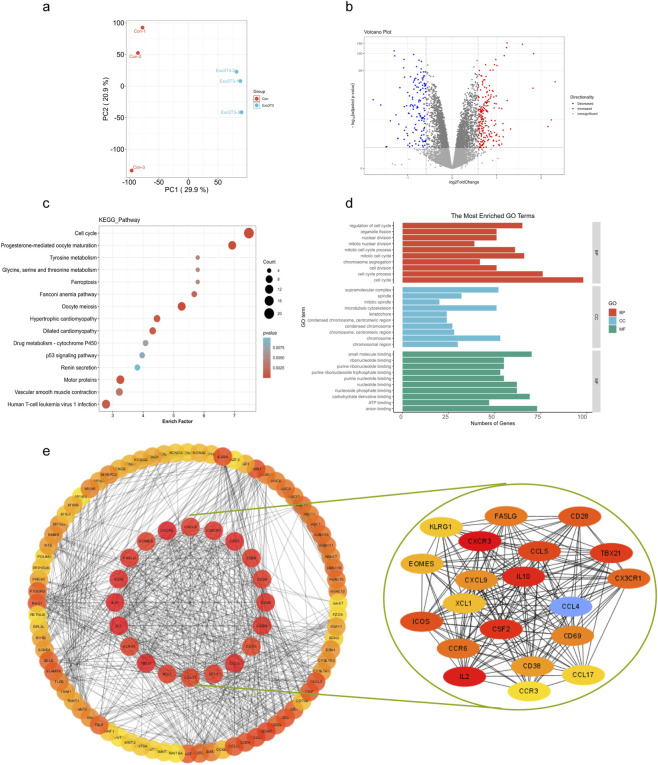
Transcriptional changes induced in cultured 3T3-L1 by BSNs treatment. **(a)** PCA plot of RNAseq. **(b)** Volcano plot of RNASeq results. **(c)** Functional annotation of DEGs was performed using KEGG pathway annotations, The KEGG pathway names are annotated on the left bar plot (RNAseq). **(d)** Functional annotation of DEGs was performed using GO enrichment analysis, The GO items’ names are annotated on the left bar plot (RNAseq). **(e)** PPI network associated with the DEGs in normal and BSNs-treated 3T3-L1 cells was constructed by STRING v 12.0. The top 50 and Top 20 core genes were shown.

To investigate whether BSNs affect transcriptome alternation, we performed RNA-seq analysis upon BSNs-treated or not-treated 3T3-L1. Principal-component (PCA) analysis revealed that BSNs-treated groups segregated from untreated groups (Figure 3a). BSNs-treated groups displayed an altered global transcription profile, including 203 upregulated and 135 downregulated DEGs with | log2(Fold Change) | >0.585 and padj < 0.05 (Figure 3b). Functional enrichment analysis of the DEGs based on the KEGG and GO analysis revealed that top-ranked canonical pathways such as Cell cycle, Tyrosine metabolism, Ferroptosis, and p53 signaling pathway, which highly associated with cancer-related pathways and apoptosis-related pathways (Figures 3c,d).

### PI3K–Akt pathway as a potential regulatory axis of BSNs

Using the Search Tool for the Retrieval of Interacting Genes/Proteins database (STRING v12.0) and Cytoscape software, we constructed the protein-protein (PPI) network associated with the DEGs with | log2(Fold Change) | >1. According to their fold change, we ranked and subjected to the top 100 proteins (Figure 3e), and found that most of the core genes in top 20 proteins, such as CXCR3, IL2, IL10, CSF2, CCL5, CD28, CX3CR1, CCR6, FASLG, CXCL9, CCL4, CCL17, CCR3, were involved in the PI3K-Akt pathway. These findings suggest that the PI3K-Akt pathway may be a key pathway through which BSNs influence cell fate.

### BSNs promote apoptosis through the Akt–p53 signaling pathway

**FIGURE 4:**
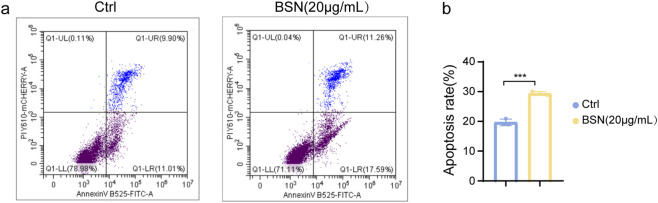
The effect of BSNs on cell apoptosis of A549 cells. **(a)** Cell apoptosis was measured by Flow cytometry. **(b)** Graphs showing quantification of apoptosis rate. This experiment was performed in triplicates, values are presented as mean ± SD. ^ns^P > 0.05, *P < 0.05, **P < 0.01, ***P < 0.001, ****P < 0.0001, compared with control.

The PI3K-Akt pathway regulates multiple aspects of cell fate. Studies have shown that p53 is regulated by the PI3K-Akt signaling pathway (Zhou et al., 2025). Activation of p53 induces cell cycle arrest and apoptosis in cells (Ryan et al., 2001; Saha et al., 2013), and referring to RNA-seq results mentioned above, we detected apoptosis in cells treated with 20 μg/mL BSNs for 24 h by flow cytometry. Early apoptosis appeared to be unaffected while late apoptotic cells were increased by ∼1.5 fold by BSNs treatment compared with the normal control group (Figures 4a,b), indicating BSNs promoted the late-stage apoptosis of A549 cells.

**FIGURE 5:**
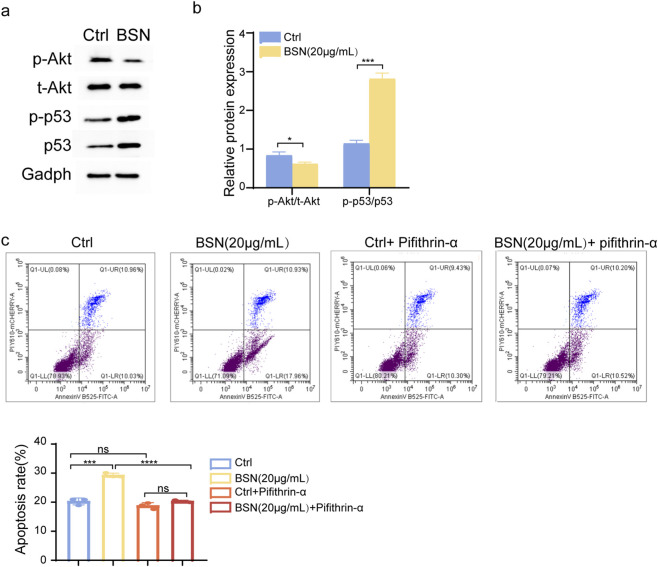
The mechanism of BSNs on cell apoptosis of A549 cells. **(a)** The protein expression levels of p-Akt, Akt, p-p53, and p53 were determined by western blot analysis. **(b)** Graphs showing quantification of **(a)**. **(c)** Cell apoptosis was measured by Flow cytometry when exposed to 10 μM Pifithrin-α for 24 h. The figure below showing quantification of apoptosis rate. All experiments were performed at least in triplicates, values are presented as mean ± SD. ^ns^P > 0.05, *P < 0.05, **P < 0.01, ***P < 0.001, ****P < 0.0001, compared with control.

To identify whether BSNs affected p53-dependent apoptosis, we examined the protein expression of p53 and phosphorylated p53 (p-p53) and its upstream factor, Akt, phosphorylated Akt (p-Akt) in normal and BSNs-treated A549 cells. As depicted in Figures 5a,b, the ratio of p-Akt/Akt was markedly decreased while p-p53/p53 was increased in the treated group compared with the normal group. Furthermore, BSNs failed to reverse the inhibitory effects of pifithrin -α, the p53 inhibitor on apoptosis, indicating that BSNs promoted apoptosis of A549 cells through p53 signaling (Figure 5c). Therefore, BSNs partially induced A549 apoptosis by association with the Akt-p53 pathway.

## Discussion

Over the last few decades, the anticancer potential of natural products has been widely investigated (Barbuti and Chen, 2015; Sarwar et al., 2020). Natural products have emerged as a promising source for anticancer drug development due to their multiple advantages, like antitumor effect, low toxicities, and rich sources. Our study attempted to elucidate the anti-cancer effects of nanoparticles derived from moso bamboo shoots in A549 Lung cancer cells. BSNs showed good activity against A549 Lung cancer cells on cell proliferation, migration, and invasion. Our RNAseq analysis speculated the Akt-p53 signaling pathway as the major pathway in response to BSNs administration. Flow cytometry analysis revealed that BSNs induced apoptosis of A549 lung cancer cells through the p53 signaling pathway.

There are multiple pieces of literature confirming the presence of exosome-like nanovesicles in plant extracts, however, further scientific investigations are needed to explore the interplay of cross-kingdom interactions. Like reports by Stefania Raimondo, citrus limon L.-derived nanovesicles have been reported to be ingested by the human chronic myeloid leukemia cell line, LAMA84, the human colorectal adenocarcinoma cell line, SW480, and the human lung carcinoma cell line, A549, induce TRAIL-medicated cell death to inhibited CML xenograft growth *in vivo* and *in vitro* (Raimondo et al., 2015). Baomei Wang, et al. reported a grapefruit-derived nanovesicle that targeted intestinal macrophages and attenuated intestinal inflammation by exerting its immunomodulation effect (Wang et al., 2014). In the present study, BSNs were identified as exosome-like nanovesicles by successful PHK26 labeling and TEM analysis. Furthermore, by immunofluorescence, BSNs internalization into A549 cells may indicate a regulatory effect on cell biological processes.

There have been several reports on the composition analysis of bamboo extracts. The leaves of bamboo contain powerful nutrient ingredients, such as natural antioxidants (like quercetin (Zhang et al., 2011), Gallic acid (Zhang et al., 2022), and Cinnamic acid (Sova, 2012)), anti-inflammatory compounds (p-Coumaric acid (Dolrahman et al., 2023) and tetrahydrocurcumin (Heidari-Soreshjani et al., 2017)), polysaccharides and minerals (Cheng et al., 2023; Ying et al., 2017). In contrast, there is a paucity of reports on the composition analysis of bamboo shoot extracts. Reda F. A. Abdelhameed isolated the ethyl acetate extracts of bamboo shoots and identified one new compound (7-hydroxy, 5-methoxy, methyl cinnamate) and four known compounds (4-keto-pinoresinol, tamarixetin, 3,4-dihydroxybenzoic acid, and methyl ferulate) (Abdelhameed et al., 2021). Of these, 4-keto-pinoresinol, a naturally NRF2/ARE activator, suppresses oxidative damage through the activation of PI3K/ART signaling (Chen et al., 2012). However, the involved mechanism in cancer needs to be studied further; tamarixetin has been found to exert a suppression effect on multiple tumor types, like live cancer cells (Xu et al., 2019), lung adenocarcinoma cells (Sak et al., 2018), and leukemia cells (Alsharairi, 2023). Our study showed a population of stable and bioactive nanovesicles in aqueous extracts of bamboo shoots which suppressed cell proliferation, migration, and invasion of A549 lung cancer cells rather than normal cells (3T3-L1) or another type of cancer cells (Hela), indicating its specific tumor targeting. Many anti-cancer drugs eventually activate a p53-mediated apoptotic pathway in cancerous cells. (Johnstone et al., 2002). Based on RNAseq analysis, we showed that the components of BSNs regulated the cell cycle, amino acid metabolism, ferroptosis, and apoptosis. Our results of WB and flow cytometry further validated that the components of BSNs induced cell apoptosis through p53 signaling. Thus, as an outlook, we speculated on the possibilities of developing BSNs as anticancer agents.

Longitudinal studies are needed in future studies. A specific identification of BSNs inclusions accountable for such activity need to be explored and the mechanism of cross-kingdom interactions to be investigated. In addition, it should be noted that findings derived from *in vitro* cell experiments have limited direct clinical translatability. Pre-clinical animal experiments are often essential for understanding the therapeutic potential of BSNs. Furthermore, though the BSNs are of edible nature with relatively safe, detailed studies on the safety and efficacy of BSNs in NSCLC are needed before recommended for clinical use.

In summary, our study demonstrated the role of BSNs in suppressing malignant cellular behaviors such as proliferation, migration, invasion, and apoptosis. It could be concluded that the nanovesicles from Bamboo shoots possess anticancer potential, which is dependent on p53 signaling. This study provides proof of a new anticancer strategy based on plant-edible nanovesicles. Further study is needed to have a specific identification of BSNs inclusions accountable for such activity and to investigate the therapeutic potential in animal or human experiments.

## Materials and methods

### BSNs extraction

Nanovesicles were extracted from moso bamboo shoots (Phyllostachys edulis) obtained from Jingshan Town, Jingshan Village, Yuhang District, Hangzhou City, Zhejiang Province, China. 225.5 g Bamboo shoots were carefully washed in water, and 500 mL bamboo shoots juice was squeezed and filtered by gauze. The juice was sequentially centrifuged at 2,000 × g for 10 min, 4 °C, and 10,000 × g for 10 min, 4 °C. The supernatant was then centrifuged at 110,000 × g for 75 min using Beckman Coulter (OptiamTM L-90 k Ultracentrifuge, United States), 4 °C. The pellet was suspended in 1 × PBS, filtered at 0.45 μm and 0.22 μm pore filter, and centrifuged at 110,000 × g for 75 min, 4 °C. The pellet was suspended in 100 μL 1 × PBS, sequentially centrifuged at 13,000 × g for 1h, 4 °C. The supernatant was deposited at the China National Bamboo Research Center (Hangzhou, China) at −80 °C.

### Transmission electron microscopy (TEM)

5 μL Nanovesicles samples were added to copper grids at room temperature for 5 min, stained with 2% uranyl acetate for 1 min, and dried for 20 min at room temperature. Transmission Electron Microscopy (Tecnai G2 Spirit BioTwin, FEI, United States) was used for detection.

### Nanoparticle tracking analysis (NTA)

Nanovesicle samples were diluted 1000-fold in 1 × PBS for NTA measurements. Nanoparticle tracking analyzer instrument (ZetaVIEW S/N 17-310, PARTICLE METRIX, Germany) was used for detection and ZetaView 8.04.02 was used for the determination of size, distribution, and concentration of nanovesicles.

### Cell culture

Human lung carcinoma (A549), cervical carcinoma (Hela), and non-cancer cells (3T3-L1 preadipocytes and mouse macrophages, RAW264.7) were purchased from Wayen Biotechnologies (Shanghai), Inc. A549, Hela and RAW264.7 were maintained in Dulbecco’s modified Eagle’s medium (DMEM; HyClone, United Kingdom) supplemented with 10% fetal bovine serum (FBS; Gibco BRL), and 1% penicillin/streptomycin (P/S) in the presence of 5% CO_2_ at 37 °C under humidified conditions. 3T3-L1 preadipocytes were cultured in DMEM high glucose (ATCC®, Cat. number 30-2002) completed medium. Cells were passaged once they reached 80% confluency. The cell medium was changed every 2 days. Among the cell lines used, A549 cells were selected as the primary experimental model, as they harbor wild-type p53 and exhibit stable Akt protein expression (Chen et al., 2017; Kawabe et al., 2000).

### Uptake of BSNs by A549 cells

To monitor the BSNs’ trafficking, nanovesicles were labeled with PKH26 Red (Sigma Aldrich, United States) for 5 min at room temperature. PKH-labeled BSNs were purified by a fluorescence adsorption column. Excessive dye was removed by 1,000 × g centrifugation for 4 min. A549 cells were grown on coverslips (NEST, China) and treated with 20 μg/mL of Purified labeled nanovesicles at 37 °C, 5%CO_2_. After 6 h of co-culture, cells were fixed and stained with Actin-Tracker Green 488 (Molecular probes, Life Technologies, United States) and nuclei were stained with DAPI (1 μg/mL) and subsequently analyzed by confocal microscopy.

### Cell viability

Cell viability was detected by CCK8 assay (Beyotime, China). Briefly, A549 (5000 cells/well) and 3T3-L1 (3000 cells/well) were seeded into 96-well plates and exposed to escalating doses of BSNs (5–20 μg/mL) for 24, 48, or 72 h. After cultured, the culture medium was replaced with 10% CCK-8 fresh medium, and the cells were incubated for 1 h at 5% CO_2_, 37 °C. The absorbance of each sample at a wavelength of 450 nm (A450) was detected by a microplate reader.

### Cell migration

Scratch wound healing assay was performed to determine cell migration. Cells (∼2 × 10^5^/mL) were plated in 12-well plates. After incubation for 24 h, wounds were scratched with a 200 μL sterile pipette tip and the cells were washed with PBS twice to remove the non-adherent debris. The cells were cultured in serum-free DMEM. The wound surface at 0 and 24 h after the start of the assay was observed by light microscope and its width was measured using ImageJ software.

### Cell invasion

Transwell migration assay was performed to determine cell invasion ability by Corning transwell insert chambers (8 mm pore size; Corning). Cells (1 × 10^5^/mL) were cultured in the upper chamber with 500 μL DMEM with 1% FBS and 1% P/S, and 500 μL DMEM with 20% FBS and 1% P/S were added to the lower chamber. After incubation for 48h, the cells in the lower chamber were fixed by 4% paraformaldehyde (PFA) and stained with 0.1% Crystal violet Stain Solution for 10 min. The imaging of the cell invasion was observed using a light microscope.

### RNA sequencing (RNAseq) analysis

3T3-L1 cells were collected for mRNA extraction and subjected to RNAseq analysis after 24 h of BSNs (20 μg/mL) co-culture. RNAseq was conducted by Wayen Biotechnologies (Shanghai), Inc. using Illumina NovaSeq 6000 platform. Differential expression genes (DEGs) (| log2(Fold Change) | >0.585 and padj < 0.05) were selected for Gene Ontology (GO) and Kyoto Encyclopedia of Gene and Genomes (KEGG) pathway analyses using the ClusterProfiler R package.

### Cell apoptosis

Cell apoptosis was measured by flow cytometry using an Annexin V-FITC/propidium iodide (PI) apoptosis detection kit (Beyotime, China) according to the manufacturer’s manual. After co-culturing with BSNs (20 μg/mL), A549 cells were stained with anti-Annexin V-FITC/PI at room temperature in the dark for 15 min cells were immediately analyzed using BD Accuri C6 Plus (BD Biosciences) and the results were calculated by FlowJo software (version 7.6.2).

### Western blot (WB) analysis

Total proteins in A549 cells co-cultured with or not with BSNs (20 μg/mL) were extracted with RIPA lysis buffer. Total proteins were electrophoresed on 10% SDS-PAGE and transferred to the PVDF membrane. After blocking with 5% non-fat milk at room temperature for 2 h, primary antibodies were incubated under 4° Covernight against p-Akt (66444-1-Ig, proteintech, United States), t-Akt (10176-2-AP, proteintech, United States), p-p53 (67826-1-Ig, proteintech, United States), p53 (10442-1-AP, proteintech, United States) and GADPH-HRP (HRP-60004, proteintech, United States). Then the membranes were washed with 1 × PBS and incubated with the second antibody at room temperature for 1 h. Protein bands were visualized using the Chemidoc Imaging system (Bio-Rad) and quantified using ImageJ (Version 1.46).

### Statistical analysis

All experiments were performed in triplicate and data are expressed as the mean ± standard deviation (SD). Statistical analysis was performed using GraphPad Prism 8 software (GraphPad Software, Inc.) and was done with an unpaired two-tailed Student's t-test. P < 0.05 was considered statistically significant.

## Data Availability

The sequencing data generated in this study have been deposited in NCBI under accession number PRJNA1444822. If any additional data support is needed, please contact the corresponding author(s) of this study.

## Supplementary material

SUPPLEMENTARY FIGURE S1 The effect of BSNs on 3T3-L1 cells, Hela cells, and raw264.7 cells. **(A)** The proliferation rate of 3T3-L1 cells under BSNs treatment was determined by CCK8 assays. **(B–D)** Scratch wound healing assay was performed to determine cell migration of 3T3-L1 cells **(B)**, Hela cells **(C)**, and raw264.7 cells **(D)**. Scale bar: 400 μm. **(E)** Graphs showing quantification of wound healing rate of 3T3-L1 cells, Hela cells, and raw264.7 cells. All experiments were performed at least in triplicates, values are presented as mean ± SD. ^ns^P > 0.05, *P < 0.05, **P < 0.01, ***P < 0.001, ****P < 0.0001, compared with control.

## References

[B1] Abdelhameed R. F. A. Habib E. S. Ibrahim A. K. Yamada K. Abdel-Kader M. S. Ahmed S. A. (2021). Chemical constituent profiling of phyllostachys heterocycla Var. pubescens with selective cytotoxic polar fraction through EGFR inhibition in HepG2 cells. Molecules 26 (4), 940. 10.3390/molecules26040940 33578916 PMC7916669

[B2] Alsharairi N. A. (2023). Quercetin derivatives as potential therapeutic agents: an updated perspective on the treatment of nicotine-induced non-small cell lung cancer. Int. J. Mol. Sci. 24 (20), 15208. 10.3390/ijms242015208 37894889 PMC10607898

[B3] Bai J. W. Qiu S. Q. Zhang G. J. (2023). Molecular and functional imaging in cancer-targeted therapy: current applications and future directions. Signal Transduct. Target Ther. 8 (1), 89. 10.1038/s41392-023-01366-y 36849435 PMC9971190

[B4] Barbuti A. M. Chen Z. S. (2015). Paclitaxel through the ages of anticancer therapy: exploring its role in chemoresistance and radiation therapy. Cancers (Basel) 7 (4), 2360–2371. 10.3390/cancers7040897 26633515 PMC4695897

[B5] Chang J. L. Chow J. M. Chang J. H. Wen Y. C. Lin Y. W. Yang S. F. (2017). Quercetin simultaneously induces G(0)/G(1) -phase arrest and caspase-mediated crosstalk between apoptosis and autophagy in human leukemia HL-60 cells. Environ. Toxicol. 32 (7), 1857–1868. 10.1002/tox.22408 28251795

[B6] Chen C. Guan X. Liu X. Zhuang W. Xiao Y. Zheng Y. (2022). Polysaccharides from bamboo shoot (leleba oldhami nakal) byproducts alleviate antibiotic-associated diarrhea in mice through their interactions with gut microbiota. Foods 11 (17), 2647. 10.3390/foods11172647 36076830 PMC9455761

[B7] Chen H. H. Chen Y. T. Huang Y. W. Tsai H. J. Kuo C. C. (2012). 4-Ketopinoresinol, a novel naturally occurring ARE activator, induces the Nrf2/HO-1 axis and protects against oxidative stress-induced cell injury *via* activation of PI3K/AKT signaling. Free Radic. Biol. Med. 52 (6), 1054–1066. 10.1016/j.freeradbiomed.2011.12.012 22245092

[B8] Chen W. Zheng R. Baade P. D. Zhang S. Zeng H. Bray F. (2016). Cancer statistics in China, 2015. CA Cancer J. Clin. 66 (2), 115–132. 10.3322/caac.21338 26808342

[B9] Chen B. Tan Y. Liang Y. Li Y. Chen L. Wu S. (2017). Per2 participates in AKT-Mediated drug resistance in A549/DDP lung adenocarcinoma cells. Oncol. Lett. 13 (1), 423–428. 10.3892/ol.2016.5430 28123577 PMC5245158

[B10] Chen Z. Jia J. Gui D. Liu F. Li J. Tu J. (2022). Functional and postoperative outcomes after high-intensity interval training in lung cancer patients: a systematic review and meta-analysis. Front. Oncol. 12, 1029738. 10.3389/fonc.2022.1029738 36741720 PMC9895778

[B11] Cheng Y. Wan S. Yao L. Lin D. Wu T. Chen Y. (2023). Bamboo leaf: a review of traditional medicinal property, phytochemistry, pharmacology, and purification technology. J. Ethnopharmacol. 306, 116166. 10.1016/j.jep.2023.116166 36649850

[B12] Dhall S. Coksaygan T. Hoffman T. Moorman M. Lerch A. Kuang J. Q. (2019). Viable cryopreserved umbilical tissue (vCUT) reduces post-operative adhesions in a rabbit abdominal adhesion model. Bioact. Mater 4 (1), 97–106. 10.1016/j.bioactmat.2018.09.002 30723842 PMC6351431

[B13] Dolrahman N. Mukkhaphrom W. Sutirek J. Thong-Asa W. (2023). Benefits of p-coumaric acid in mice with rotenone-induced neurodegeneration. Metab. Brain Dis. 38 (1), 373–382. 10.1007/s11011-022-01113-2 36308586

[B14] He M. X. Wang J. L. Qin H. Shui Z. X. Zhu Q. L. Wu B. (2014). Bamboo: a new source of carbohydrate for biorefinery. Carbohydr. Polym. 111, 645–654. 10.1016/j.carbpol.2014.05.025 25037399

[B15] Heidari-Soreshjani S. Asadi-Samani M. Yang Q. Saeedi-Boroujeni A. (2017). Phytotherapy of nephrotoxicity-induced by cancer drugs: an updated review. J. Nephropathol. 6 (3), 254–263. 10.15171/jnp.2017.41 28975109 PMC5607991

[B16] Johnstone R. W. Ruefli A. A. Lowe S. W. (2002). Apoptosis: a link between cancer genetics and chemotherapy. Cell. 108 (2), 153–164. 10.1016/s0092-8674(02)00625-6 11832206

[B17] Jongrungraungchok S. Madaka F. Wunnakup T. Sudsai T. Pongphaew C. Songsak T. (2023). *In vitro* antioxidant, anti-inflammatory, and anticancer activities of mixture Thai medicinal plants. BMC Complement. Med. Ther. 23 (1), 43. 10.1186/s12906-023-03862-8 36765341 PMC9912591

[B18] Kalwani N. Remenschneider A. K. Faquin W. Ferry J. Holbrook E. H. (2015). Plasmacytoma of the clivus presenting as bilateral sixth nerve palsy. J. Neurol. Surg. Rep. 76 (1), e156–e159. 10.1055/s-0035-1554930 26251795 PMC4520983

[B19] Kawabe S. Roth J. A. Wilson D. R. Meyn R. E. (2000). Adenovirus-mediated p16INK4a gene expression radiosensitizes non-small cell lung cancer cells in a p53-dependent manner. Oncogene 19 (47), 5359–5366. 10.1038/sj.onc.1203935 11103937

[B20] Li X. Lin Z. Zhang B. Guo L. Liu S. Li H. (2016). beta-elemene sensitizes hepatocellular carcinoma cells to oxaliplatin by preventing oxaliplatin-induced degradation of copper transporter 1. Sci. Rep. 6, 21010. 10.1038/srep21010 26867799 PMC4751482

[B21] Li Q. Wu W. Fang X. Chen H. Han Y. Liu R. (2022). Structural characterization of a polysaccharide from bamboo (Phyllostachys edulis) shoot and its prevention effect on colitis mouse. Food Chem. 387, 132807. 10.1016/j.foodchem.2022.132807 35397273

[B22] Pilut C. N. Manea A. Macasoi I. Dobrescu A. Georgescu D. Buzatu R. (2022). Comparative evaluation of the potential antitumor of Helleborus purpurascens in skin and breast cancer. Plants (Basel) 11 (2), 194. 10.3390/plants11020194 35050083 PMC8779569

[B23] Raimondo S. Naselli F. Fontana S. Monteleone F. Lo Dico A. Saieva L. (2015). Citrus limon-derived nanovesicles inhibit cancer cell proliferation and suppress CML xenograft growth by inducing TRAIL-Mediated cell death. Oncotarget 6 (23), 19514–19527. 10.18632/oncotarget.4004 26098775 PMC4637302

[B24] Ramakanth G. S. Uday Kumar C. Kishan P. V. Usharani P. (2016). A randomized, double blind placebo controlled study of efficacy and tolerability of withaina somnifera extracts in knee joint pain. J. Ayurveda Integr. Med. 7 (3), 151–157. 10.1016/j.jaim.2016.05.003 27647541 PMC5052364

[B25] Ramalingam S. S. Owonikoko T. K. Khuri F. R. (2011). Lung cancer: new biological insights and recent therapeutic advances. CA Cancer J. Clin. 61 (2), 91–112. 10.3322/caac.20102 21303969

[B26] Ryan K. M. Phillips A. C. Vousden K. H. (2001). Regulation and function of the p53 tumor suppressor protein. Curr. Opin. Cell. Biol. 13 (3), 332–337. 10.1016/s0955-0674(00)00216-7 11343904

[B27] Saha M. N. Qiu L. Chang H. (2013). Targeting p53 by small molecules in hematological malignancies. J. Hematol. Oncol. 6, 23. 10.1186/1756-8722-6-23 23531342 PMC3614876

[B28] Sak K. Lust H. Kase M. Jaal J. (2018). Cytotoxic action of methylquercetins in human lung adenocarcinoma cells. Oncol. Lett. 15 (2), 1973–1978. 10.3892/ol.2017.7466 29399199 PMC5774546

[B29] Sarwar M. S. Xia Y. X. Liang Z. M. Tsang S. W. Zhang H. J. (2020). Mechanistic pathways and molecular targets of plant-derived anticancer ent-Kaurane diterpenes. Biomolecules 10 (1). 10.3390/biom10010144 31963204 PMC7023344

[B30] Sha A. Luo Y. Xiao W. He J. Chen X. Xiong Z. (2024). Plant-derived exosome-like nanoparticles: a comprehensive overview of their composition, biogenesis, isolation, and biological applications. Int. J. Mol. Sci. 25 (22), 12092. 10.3390/ijms252212092 39596159 PMC11593521

[B31] Sova M. (2012). Antioxidant and antimicrobial activities of cinnamic acid derivatives. Mini Rev. Med. Chem. 12 (8), 749–767. 10.2174/138955712801264792 22512578

[B32] Taron M. Rosell R. Felip E. Mendez P. Souglakos J. Ronco M. S. (2004). BRCA1 mRNA expression levels as an indicator of chemoresistance in lung cancer. Hum. Mol. Genet. 13 (20), 2443–2449. 10.1093/hmg/ddh260 15317748

[B33] Wang B. Zhuang X. Deng Z. B. Jiang H. Mu J. Wang Q. (2014). Targeted drug delivery to intestinal macrophages by bioactive nanovesicles released from grapefruit. Mol. Ther. 22 (3), 522–534. 10.1038/mt.2013.190 23939022 PMC3944329

[B34] Wu W. Hu J. Gao H. Chen H. Fang X. Mu H. (2020). The potential cholesterol-lowering and prebiotic effects of bamboo shoot dietary fibers and their structural characteristics. Food Chem. 332, 127372. 10.1016/j.foodchem.2020.127372 32615381

[B35] Xiong Y. Zhao Q. Gu L. Liu C. Wang C. (2018). Shenqi fuzheng injection reverses cisplatin resistance through Mitofusin-2-Mediated cell cycle arrest and apoptosis in A549/DDP cells. Evid. Based Complement. Altern. Med. 2018, 8258246. 10.1155/2018/8258246 30410558 PMC6206574

[B36] Xu J. Cai X. Teng S. Lu J. Zhou Y. Wang X. (2019). The pro-apoptotic activity of tamarixetin on liver cancer cells *via* regulation mitochondrial apoptotic pathway. Appl. Biochem. Biotechnol. 189 (2), 647–660. 10.1007/s12010-019-03033-x 31093908

[B37] Yi Q. Xu Z. Thakur A. Zhang K. Liang Q. Liu Y. (2023). Current understanding of plant-derived exosome-like nanoparticles in regulating the inflammatory response and immune system microenvironment. Pharmacol. Res. 190, 106733. 10.1016/j.phrs.2023.106733 36931541

[B38] Ying C. Mao Y. Chen L. Wang S. Ling H. Li W. (2017). Bamboo leaf extract ameliorates diabetic nephropathy through activating the AKT signaling pathway in rats. Int. J. Biol. Macromol. 105 (Pt 3), 1587–1594. 10.1016/j.ijbiomac.2017.03.124 28359892

[B39] Zhang M. Swarts S. G. Yin L. Liu C. Tian Y. Cao Y. (2011). Antioxidant properties of quercetin. Adv. Exp. Med. Biol. 701, 283–289. 10.1007/978-1-4419-7756-4_38 21445799

[B40] Zhang W. Zeng Q. M. Tang R. C. (2022). Gallic acid functionalized polylysine for endowing cotton fiber with antibacterial, antioxidant, and drug delivery properties. Int. J. Biol. Macromol. 216, 65–74. 10.1016/j.ijbiomac.2022.06.186 35788001

[B41] Zhou T. T. Li L. Guo T. H. Wang Y. H. Sun D. D. Tan J. N. (2025). Pro-apoptosis effects of yangzheng-xiaoji capsules in hepatocellular carcinoma: activation of the p53-Induced apoptotic pathway and inhibition of the PI3K/Akt pathway. Integr. Cancer Ther. 24, 15347354251352848. 10.1177/15347354251352848 40886068 PMC12399825

