# Supplementary figures and images for: Bamboo shoots-derived nanovesicles (BSNs) induce apoptosis in non-small-cell lung cancer A549 cells through the p53 signaling pathway
Source: Front Mol Biosci. 2026 Apr 9;13:1759968. doi: 10.3389/fmolb.2026.1759968 (PMC13102564; doi:10.3389/fmolb.2026.1759968)

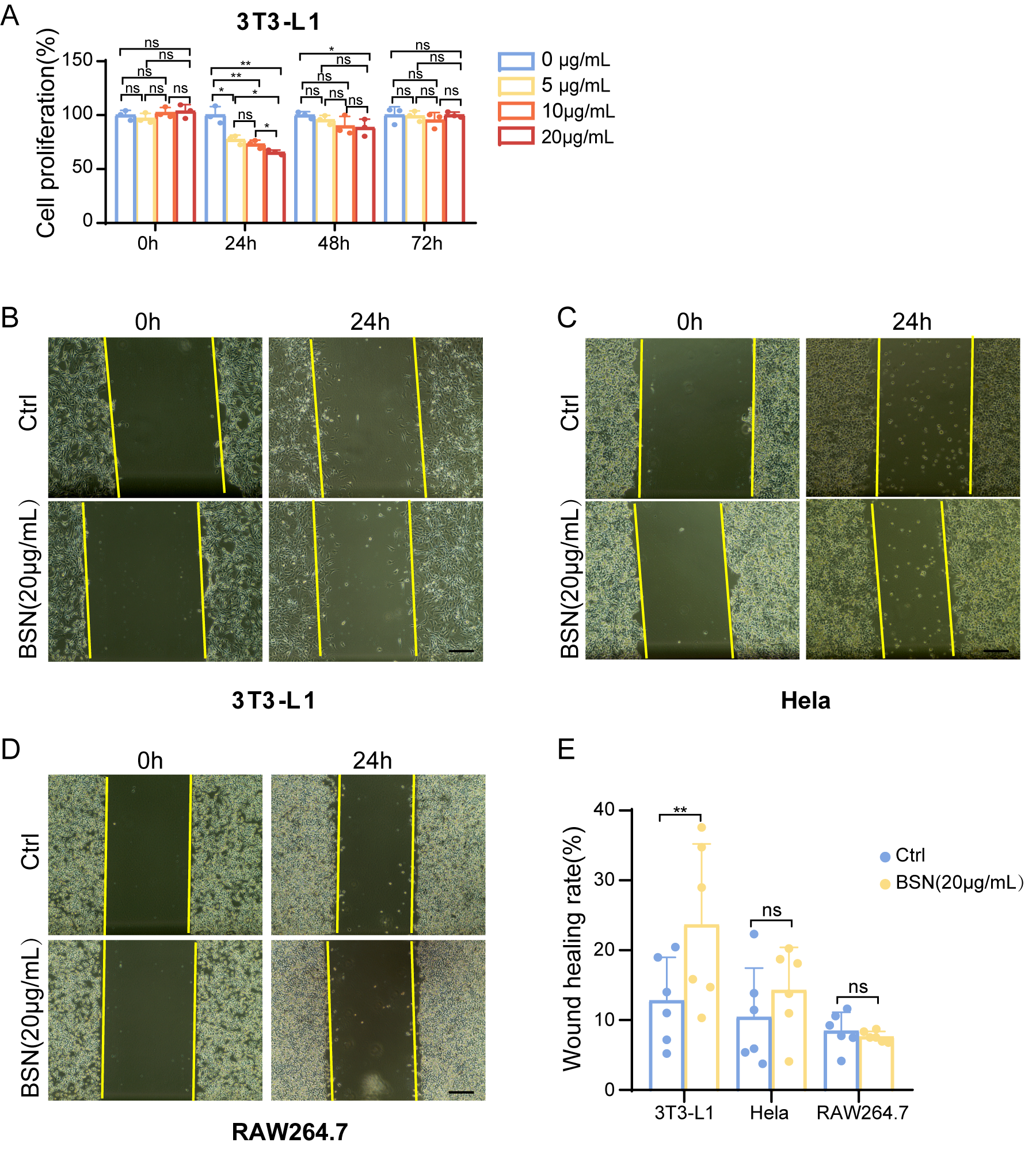

Supplement: Supplementary file 1 [file Image1.tif]
